# Advances in Small Angle Neutron Scattering on Polysaccharide Materials

**DOI:** 10.3390/polym16040490

**Published:** 2024-02-09

**Authors:** Anastasiia Fanova, Konstantinos Sotiropoulos, Aurel Radulescu, Aristeidis Papagiannopoulos

**Affiliations:** 1 Forschungszentrum Jülich GmbH, Jülich Centre for Neutron Science (JCNS) at Heinz Maier-Leibnitz Zentrum (MLZ), Lichtenbergstraße 1, 85747 Garching, Germany; a.fanova@fz-juelich.de (A.F.); a.radulescu@fz-juelich.de (A.R.); 2 Hyad Mike, Nutritional Supplements Manufacturing Company, Gennadiou 1-5, 12131 Athens, Greece; kwstas_sw@hotmail.com; 3 Theoretical and Physical Chemistry Institute, National Hellenic Research Foundation, 48 Vassileos Constantinou Avenue, 11635 Athens, Greece

**Keywords:** small angle scattering, contrast variation, hierarchy, biopolymers, proteins

## Abstract

Polysaccharide materials and biomaterials gain the focus of intense research owing to their great versatility in chemical structures and modification possibilities, as well as their biocompatibility, degradability, and sustainability features. This review focuses on the recent advances in the application of SANS on polysaccharide systems covering a broad range of materials such as nanoparticulate assemblies, hydrogels, nanocomposites, and plant-originating nanostructured systems. It motivates the use of SANS in its full potential by demonstrating the features of contrast variation and contrast matching methods and by reporting the methodologies for data analysis and interpretation. As these soft matter systems may be organized in multiple length scales depending on the interactions and chemical bonds between their components, SANS offers exceptional and unique opportunities for advanced characterization and optimization of new nanostructured polysaccharide materials.

## 1. Introduction

Polysaccharide-based materials are attractive for applications in food science, biomedicine, and composite materials as they are biocompatible, abundant, and renewable [1]. The hydrophilic, nontoxic, and biodegradable characteristics of polysaccharides allow them to be incorporated in multifunctional materials with tunable properties. Self-organized and enzymatically or ionically crosslinked hydrogels have been developed by oppositely charged polysaccharides with potential in antimicrobial food packaging [2] and cartilage tissue engineering [3]. Hybrid systems from combinations of polysaccharides with inorganic nanoparticles are promising for advanced applications for therapy, diagnosis, and bioimaging [4]. Polysaccharide nanoparticles are certainly going to play a pivotal role as nanocarriers of biosubstances, emulsion stabilizers, and additives for reinforcement [5].

Polysaccharide systems are soft macromolecular materials and as such they have complex and hierarchically organized nanostructure at multiple length scales. Small angle neutron scattering (SANS) is a powerful method to investigate macromolecular conformations, self-assembly, and hierarchical morphology noninvasively, with great resolution at the nanoscale (1–1000 nm) [6]. Moreover, the advantage of neutrons over X-rays is the ability to vary the scattering contrasts between different constituents of a hydrocarbon sample over a broad range by H/D substitution. Since the molecules affected by H/D exchange are chemically the same, the physical chemistry of the samples is only marginally modified, if at all. This feature allows for the investigation of polysaccharide materials that contain multiple components, e.g., in chitosan-stabilized O/W nanoemulsions for nasal delivery [7], glucose-reinforced alginate hydrogels [8], and electrostatic assemblies between hyaluronan and surfactants [9].

This review demonstrates the capabilities of SANS in the characterization of nanostructured materials. It comprehensively presents the principles of the method and highlights the special features of contrast variation and contrast matching. It discusses recent developments in nanomaterials that include nanoparticulate systems, e.g., nanoparticles and vesicular nanocontainers, macromolecular networks, e.g., polysaccharide-based hydrogels, nanocomposites, e.g., organic-inorganic hybrids and natural and renewable materials, e.g., plant nanostructured materials. This article illustrates the advantages of SANS in the field of advanced physicochemical characterization of complex materials and biomaterials and the opportunities that arise for better understanding and optimization of the produced nanosystems.

## 2. Small Angle Neutron Scattering: Fundamentals and Experimental Practices

**Figure 1:**
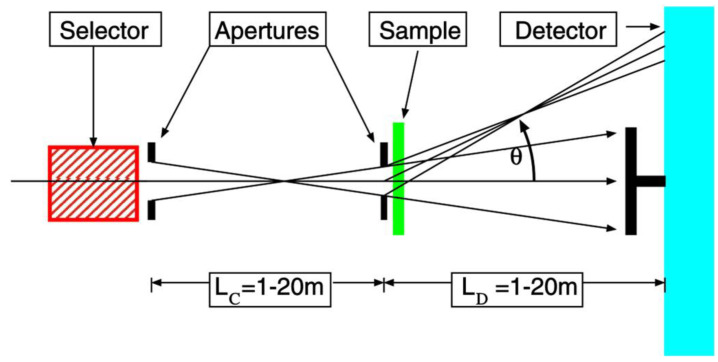
Principle of pinhole SANS diffractometry; a neutron beam entering the instrument from the left is monochromatized (by a velocity selector), collimated over a variable distance (by an adaptive system of apertures), scattered on the sample, and detected on a two-dimensional position-sensitive detector. The part of the beam that passes through the sample is collected on a beam stop in the center of the detector, which corresponds to the size of the beam divergence defined by the collimation system and thus determines the resolution (minimum detected scattering angle) of the setup. As the SANS instruments are designed for the detection of neutrons at small and very small scattering angles, they are normally very long, with variable collimation and detection length.

Comprehensive overviews on the fundamentals of scattering and the practical applications of small-angle scattering techniques across various fields, including hard matter, soft matter, and structural biology, can be found elsewhere [10,11,12,13,14,15,16]. Instead of duplicating existing content, this section aims to provide a concise introduction to the fundamentals and experimental practices. Understanding the fundamentals and benefits of the SANS technique for characterizing complex materials and biomaterials can aid in optimizing experiments and comprehending the obtained results. In general, scattering techniques explore matter in reciprocal space, and the information about the microdynamics and microstructure of the studied sample from a neutron scattering experiment is contained in the neutron intensity, which is a function of the energy transfer ℏω  and the momentum transfer ℏQ. Elastic scattering with neutrons provides structural evidence about the arrangement of atoms and molecules or the magnetic moments, which delivers information about the size and number density of the scattering objects as well as their orientation and the correlation between them. This information is contained in the neutron intensity measured as a function of the wavevector transfer Q, where Q = (4π/λ) sinθ/2 is the modulus of the scattering vector $\vec{Q}$, with λ—the neutron wavelength, and θ—the scattering angle (Figure 1). Q acts as a kind of reverse yardstick: large Q values refer to short distances, while a small Q corresponds to large objects. Thus, crystalline arrangements of atoms and molecules, typically characterized by sizes and correlations of a few Å, are studied by analyzing scattering at large angles and/or using neutrons with short wavelengths, typically λ < 2 Å. This is the case with neutron diffraction (ND) or wide-angle neutron scattering (WANS) techniques. On the other hand, associations of molecules or macromolecules that lead to large-scale objects such as micelles, vesicles, or general phase separation in a sample, with a characteristic size in the range of a few tens to a thousand Å, are characterized by observing scattering at small angles (SANS, Figure 1) using neutrons with a long wavelength, typically λ > 3 Å.

**Figure 2:**
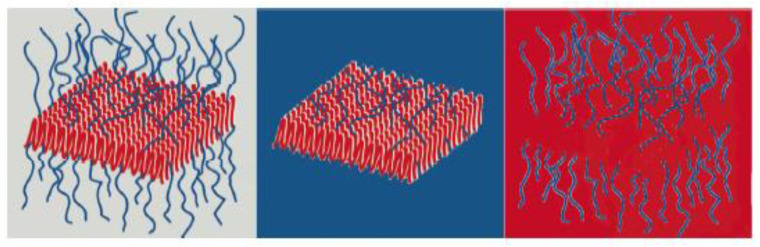
Example of contrast matching between different domains of a two-dimensional core-brush micelle formed by a crystalline-amorphous diblock copolymer and the solvent: by modification of the solvent and/or one of polymer blocks SLD the full contrast, core contrast, and brush contrast conditions (from left to right) can be achieved [17].

Neutrons interact with matter via short-range nuclear interactions and therefore see the nuclei in a sample rather than the diffuse electron cloud observed with X-rays. In magnetic samples, neutrons are scattered by the magnetic moments associated with unpaired electron spins (dipoles). In contrast to X-rays, neutrons “see” light atoms in the presence of heavier ones and can distinguish neighboring elements more easily. Since the cross section of neutron scattering generally varies between isotopes of the same element, exploiting the isotope substitution method can allow neutrons to highlight structural and dynamic details in a complex multicomponent compound (Figure 2). In particular, the strong difference in cross section between hydrogen isotopes protium (^1^H) and deuterium (^2^H) is of great importance for the study of hydrocarbon materials, such as synthetic and natural polymers, using contrast variation technique by involving D-labeling of macromolecules.

For a sample with N identical scattering particles of volume V_p_, located at random positions and in random orientations, NV_p_ = ϕV_sample_, where ϕ is the volume fraction occupied by the scattering particles in the sample with volume V_sample_. The particles are characterized by a scattering length density (SLD) *ρ*_p_, which is defined as

(1) $$\rho_{p}=\frac{\sum_{i}^{atoms\;in\;p}b_{i}}{V_{p}}$$

where the sum of all scattering length contributions b_i_ of the nuclei in the particle is taken and V_p_ is the volume of the particle. If the particles are macromolecules, V_P_ represents the molar volume of a macromolecule V_m_ = M_w_/(N_A_ ζ), where M_w_ is the molecular weight of the macromolecule, N_A_ is the Avogadro number, and ζ is the bulk density of the material. The contribution of these particles to the small angle scattering intensity I(Q) is defined by

(2) $$I\left(Q\right)=d\Sigma /d\Omega \left(Q\right)=\phi \Delta \rho^{2}\;V_{p}P\left(Q\right)S\left(Q\right)+Bckgd$$

where dΣ/dΩ(Q) is the coherent macroscopic scattering cross section of the particles, P(Q) is the particle form factor, which corresponds to the intra-particle correlations, and S(Q) is the structure factor, which denotes the inter-particle correlation effects. The contrast Δ*ρ* = *ρ*_p_ − *ρ*_env_ is the difference between the SLD of the scattering particles *ρ*_p_ and their environment *ρ*_env_, where the environment can be a solvent or a matrix.

Typically, the factor (ϕ Δ*ρ*^2^ V_p_) is referred to as the “forward scattering” I_0_ from the ensemble of scattering particles. When determined experimentally, it can lead to the evaluation of various parameters such as the M_w_ or the ϕ of the particles in the sample or the *ρ*_p_. Notably, the determination of any one of these values is contingent upon having knowledge of the other two parameters beforehand, either through other types of analyses or from the sample preparation/synthesis procedure. The term Bckgd stands for a constant background, which mainly results from the featureless incoherent scattering contribution of the sample components and can be observed as a constant level at high Q. Neutron scattering is characterized by coherent and incoherent scattering contributions, each of which depends on the scattering properties of the individual nuclei (Table 1). Coherent scattering is characterized by the coherent scattering cross section σ_coh_ and produces interferences that provide information about the structure of the scattering system revealed by the Q-dependent scattering features observed in I(Q), while incoherent scattering, characterized by σ_inc_, contains no structural information and appears in a SANS experiment only as a Q-independent constant background.

The scattering cross sections and scattering lengths of various elements and isotopes relevant to the present study are listed in Table 1.

**Figure 3:**
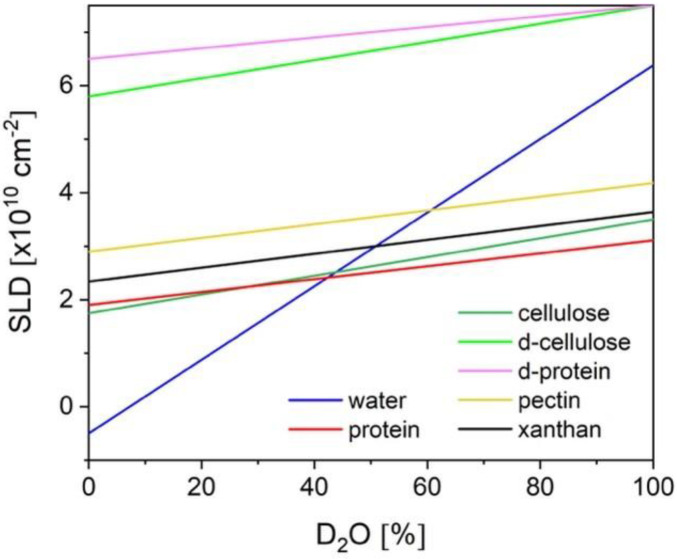
Variations of SLD as a function of solvent composition corrected for exchangeable protons in different polysaccharides and proteins in different forms; fully deuterated proteins and cellulose are specifically indicated. The SLDs of cellulose taken from [19], of pectin from [20], and of xanthan from [21].

The SLD of a compound can be calculated for its known composition according to Equation (1), taking into account the coherent scattering lengths b_coh_, which are usually tabulated for each known nucleus. Since the coherent scattering lengths b_H_, b_D_, and b_C_ vary widely (Table 1), the substitution of H/D allows large variations of Δ*ρ* between hydrocarbon materials and their environment. This can be achieved either by complete or partial deuteration of the environment (solvent), which represents the so-called external contrast variation, or by deuteration of selected parts of the hydrocarbon sample (polymer blocks, head or tails of phospholipids, etc.), which is the method of internal contrast variation. Figure 3 shows at which composition H_2_O/D_2_O of the solvent the scattering contribution from different polysaccharides and proteins can be matched out in a SANS experiment.

The contrast matching method is particularly useful for the unambiguous structural characterization of multicomponent systems such as polysaccharide-protein complexes formed in a solution. The internal contrast variation may be applied by using partially deuterated proteins or polysaccharides for matching out their scattering contribution in 100% D_2_O. For a ternary system containing a water buffer, polysaccharide, and protein, for example, with a water buffer (solvent) as a reference, the macroscopic cross-section in terms of partial scattering functions of the components assumes the following form:

(3) $$\frac{d\Sigma }{d\Omega }\left(Q\right)={\left(\rho_{ps}-\rho_{buf}\right)}^{2}P_{ps-ps}\left(Q\right)+\left(\rho_{ps}-\rho_{buf}\right)\left(\rho_{prot}-\rho_{buf}\right)P_{ps-prot}\left(Q\right)+{\left(\rho_{prot}-\rho_{buf}\right)}^{2}P_{prot-prot}\left(Q\right)$$

Here, the subscripts “ps” and “prot” indicate polysaccharide and protein, respectively. The partial scattering functions contain structural information about the polysaccharide and protein conformation within the complexes and their mutual interaction. Particularly, the cross term that corresponds to the polysaccharide–protein interaction and varies in shape and sign can offer valuable information about the nature of interactions between the two components and thus their cooperative assembling: a negative P_ps-prot_(Q) indicates repulsive interactions between polysaccharide and protein, while attractive interactions are denoted by a positive cross term. They partial scattering functions may be accessed separately by proper variation of the buffer scattering length density ρ_buf_, thereby varying the contrast factors in Equation (3).

**Figure 4:**
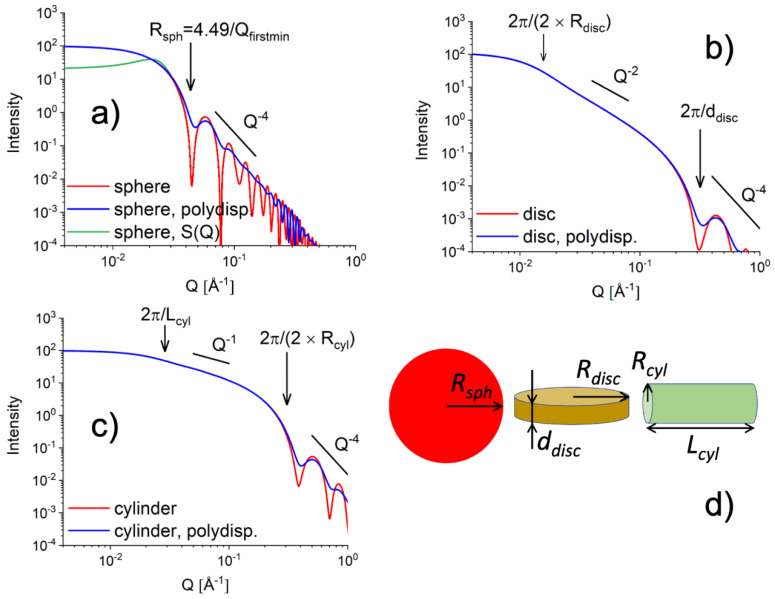
The form factor of objects with different shapes, such as (**a**) spheres, (**b**) disks, and (**c**) cylinders, is shown without (red curves) and with (blue curves) polydispersity effect in size (σ_0_ of R_sph_, d_disc_, or R_cyl_ as discussed in [23]). The occurrence of the structure factor S(Q) effect in the scattering from an ensemble of spheres is also shown (green curve in panel (**a**)). The power-law behavior of the scattered intensity in different Q regimes and the scattering features representing the main structural parameters of the objects (**d**) are also indicated in panels (**a**–**c**)). The form factors were calculated for R_sph_ = 100 Å, R_disc_ = 200 Å, d_disc_ = 20 Å, L_cyl_ = 200 Å, R_cyl_ = 10 Å, σ_0_ = 0.1 with a forward scattering I_0_ = 200 a.u., while the structure factor S(Q) [22] was evaluated for the same sphere size and a hard-sphere radius R_h_ = 120 Å at a concentration of hard-spheres in solution η = 0.2.

A collection of form factors P(Q) describing different morphologies typically formed by soft matter and biophysical systems can be found in [22]. The form factors describing scattering objects with simple geometry such as spherical, cylindrical or disc morphologies are shown in Figure 4. The correlation effects that occur in concentrated systems or the effect of polydispersity in size on the scattering patterns are also depicted.

Realistic morphologies and structures that occur in polysaccharide-based materials are more complex than the simple scattering objects shown in Figure 4. However, through skillful chemistry in sample preparation protocols and smart definition of experimental conditions in terms of neutron scattering contrast to render selected components in a complex sample as “visible” or “invisible”, the analysis of scattering data can be directed towards the possibility of applying the form factor and structure factor approach as introduced above and described in various papers on this topic, as it will be discussed in the following chapters.

## 3. Application of Small Angle Neutron Scattering on Nanostructured Polysaccharide Materials

### 3.1. Nano-Particulate Systems

Nano-particulate systems refer to systems composed of particles in the nanometer size range in at least one dimension, which is within the resolution of SANS. These systems have gained significant attention in various fields, including medicine, materials science, and electronics, due to their unique properties and potential applications. In materials science, nanoparticulate systems are employed to create materials with enhanced properties. For instance, nanoparticles can improve the mechanical strength, conductivity, or catalytic activity of materials. In medicine, nanoparticulate systems are considered powerful tools in drug delivery. Specifically, nanoparticles can be designed to encapsulate drugs and deliver them to specific target sites in the body. This allows for controlled and sustained release of the therapeutic agent, improving drug efficacy and minimizing side effects. Examples of nanoparticulate drug delivery systems include diverse morphologies and structures such as liposomes, micelles, vesicles, and polymeric nanoparticles. In this section, we considered several examples of polysaccharide containing nano-particulate systems based on their structural features and applications.

**Figure 5:**
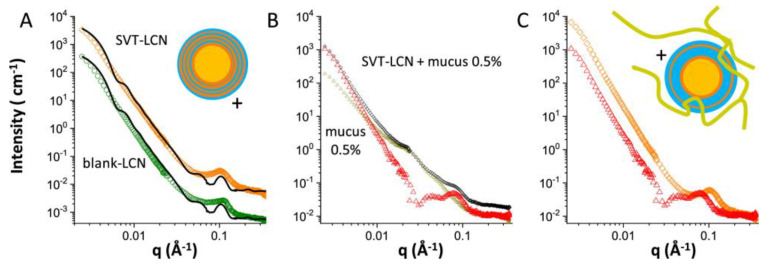
SANS intensity spectra of lecithin/chitosan nanoparticles (8 mg·mL^−1^): (**A**) spectra of blank LCNP (green circles, down-shifted for better visibility) and SVT-LCN (orange diamonds) nanoparticles together with the fit to a spherical core multilamellar shell model (continuous line); (**B**) spectra of SVT-LCN dispersed in simulated nasal mucus (porcine mucin 0.5% *w*/*v* in SNES) (black diamonds), simulated nasal mucus alone (dark-yellow triangles), and spectrum obtained by subtracting the mucus contribution from the spectrum of nanoparticles in mucus (red triangles); (**C**) spectra LCNPs nanoparticles before (orange diamonds) and after (red triangles) interaction with mucus are reported together for better visual comparison. Reprinted from [7]. Licensed under CC-BY 4.0.

Nanoparticles show promise as effective mediators in the nasal and brain delivery of bioactive compounds. However, the therapeutic efficacy depends on the ability of nanoparticles to overcome biological barriers. Three types of nanoformulations were tested to assess the delivery ability of the model hydrophobic drug simvastatin across the nasal epithelium. All three types of the investigated NPs encapsulated the drug in an oil phase surrounded by hydrophilic shells. In the first case, the oily core of the NPs was stabilized by lecithin in combination with chitosan (LCNPs). The SANS profiles of the nanostructures were modeled by the form factor of spherical multilayer vesicles with an oily core. The characteristic peak at the scattered intensity at Q ~0.1 Å^−1^ revealed the multilamellar structure which had a core-size of 180–200 nm and an interlayer spacing 6 nm (Figure 5). The second type of NPs were based on the surfactant polysorbate 80 and the anionic polyelectrolyte poly-ε-caprolactone (PCL-P80). This system formed an oily core (120–160 nm) surrounded by a raspberry-like spherical shell decorated by PCL globular clusters 10 nm in size. In the last NPs (PCL-SCH), the polysaccharide surfactant sodium caproyl hyaluronate was used instead of P80. They formed core-shell particles with core diameter larger than 200 nm and uniform polymeric shell. The sizes obtained by SANS agreed with the ones from DLS. Zeta potential measurements showed as expected that LCNPs were positively and PCL-P80/SCH were negatively charged. The surface and internal structure of the NPs defined the drug release kinetics and transport. All three cases showed better transport than the one of standard formulations which was caused by two different mechanisms. LCNPs showed strong mucoadhesion which facilitated fast permeation of the drug through the mucous. PCL-P80 and PCL-SCH provided drug permeation by uptake and penetration of the whole NPs by the nasal mucosa [7].

In another work, the mucopenetrating properties of O/W emulsions coated with PEGylated surfactants were also evaluated by the interaction with pig gastric mucin saline simulated nasal fluid. The SANS and SAXS profiles of the individual nanoemulsions and pig gastric mucin were modeled by core-shell structures (~200 nm) and the combination of flexible polypeptide backbone with the scattering from polydisperse interacting globules (~10 nm), respectively. It was shown that although incorporation of chitosan to the nanoemulsions induced attractive interactions with mucin it facilitated their diffusion. The interaction affected mucin’s spatial arrangements by creating density inhomogeneities and increasing the mesh pore size i.e., the characteristic distance between the hydrophobic globules [24].

Electrostatic complexes of sodium hyaluronate with O/W microemulsions stabilized by tetradecyldimethylamine oxide and tetradecyltrimethylammonium bromide (cosurfactant) were shown to arrange as rigid elongated structures. The SANS data from the complexes with the more flexible polyelectrolyte carboxymethyl cellulose and the polysaccharide carboxymethyl cellulose were also taken into consideration. As the cylindrical form factors did not adequately fit the data, a Monte Carlo-based approach with curved chains of droplets was applied to evaluate the persistence length and the other structural parameters [25]. Morphological transitions in didecyldimethylammonium bromide (DDAB) vesicles and hybrid vesicles of DDA/HA under the influence of BSA at pH 7 were studied by SANS. Unilamellar nanovesicles had a size of 6–8 nm and a lamellar thickness of 1.5–2.0 nm. The interaction with BSA was more intense when HA was added to the vesicular interface even if in that case both the vesicles and the protein were negatively charged. At high added BSA, the HA-decorated vesicles turned from unilamellar to multilamellar (70–90 nm) which was revealed by characteristic SANS peaks. Monte Carlo optimization combined with Bayesian inference provided the parameters, their uncertainties and their interdependencies. These findings demonstrated that the loading capacity of vesicles can be greatly enhanced by addition of polysaccharides and the resulting increase in the number of bilayers. The sustainable release properties of the vesicles are also expected to be tuned by their lamellarity [26].

Another novel delivery therapy approach is based on mRNA pharmaceuticals with applications in tumor therapy and cancer vaccination, respectively. In this case, nano-particulate formulations deliver mRNA to the target sites, where genetic transfection occurs in the cells. Polymeric systems containing condensed or/and complexed gene RNA through the electrostatic interactions are named polyplexes. Nano-polyplexes of diethylaminomethyl (DEAE)-dextran with mRNA were prepared at various molar ratios and their transfection activity on dendritic cells from human blood was assessed. The hydrodynamic radius (~100 nm) showed a strong decrease caused by aggregation at N/P ratio (amine to phosphate groups ratio) about 1 whereas the zeta potential changed from negative to positive. Increasing N/P the amount of cationic polymer in the polyplexes was found to increase around N/P~1 from 0 to about 10% while the accessible mRNA groups decreased with a pronounced minimum at the same N/P. Interestingly, transfection efficacy was higher at high N/P ratios. SAXS measurements showed that strong aggregation took place at N/P around 1 as it was seen by DLS and hierarchical structures of polyplexes and free dextran at higher molar ratios. SANS measurements were performed at different H_2_O/D_2_O ratios. In the dextran-matched solvent (at N/P~8 where free dextran is at low percentage), the conformation of mRNA was found to be different than the one of free mRNA in H_2_O. This was attributed to the interaction and complexation of the two macromolecules. However, the SANS profiles of the two biopolymers extracted by contrast matching were similar indicating that their conformations and arrangements were uniform within the polyplexes. These findings showed that the complex morphology of the studied polyplexes was related to the biological activity of the system [27].

Moreover, liposomes structures could be effectively utilized for the target drug delivery purposes due to the structural similarity between lipid bilayer and cell membrane. Additionally, liposomes offer a significant advantage by encapsulating both hydrophilic and hydrophobic drugs and directing them to the specific diseased site. Liposomes of various surface properties were studied in entangled hyaluronic acid (HA) solutions to understand the interactions of nanocarriers of drugs with biological fluids. Positively charged and PEGylated liposomes created clusters due to depletion. Cationic liposomes better separated within the HA matrix. The loading of a hydrophobic drug did not alter the morphology of the liposomes. Clustering and hard-core interactions between liposomes were readily revealed by SANS as the scattering curves deviated from the ones of vesicles at low Q. The Guinier plateau gave place to a power-law with characteristic exponents of mass fractals and correlation peaks of hard-spheres interactions [28]. HA has been also studied in combination with vesicles of dipalmitoylphosphatidylcholine (DPPC) with SANS. The scope of this work was to model the behavior of the surfactant and the polysaccharide in the synovial fluid. Since in osteoarthritis conditions, the molar mass and concentration of HA is decreased, the authors proposed an investigation of these two parameters. HA/DPPC vesicle complexes were modeled as thin spherical shell with a radius of 34–39 nm and a thickness of 6–9 nm and a polydispersity index of 0.4. The vesicular shell shrank weakly with HA concentration and the radius went through a minimum at 1 mg mL^−1^ HA for high molar mass HA. For low molar mass HA, the radius passed through a maximum at 1 mg mL^−1^ however the shell thickness decreased more strongly than for high molar mass HA up to 0.33 mg mL^−1^ and increased for higher HA concentrations. In addition, DLS measurements revealed that high molar mass HA induced aggregation of the vesicles. The scattering experiments were complemented by adsorption studies on gold where high molar mass HA resulted in thick and amorphous films which offer high load bearing and resistance to wear. Based on the findings the authors proposed that low molar mass HA penetrated the lipid bilayers while high molar mass HA absorbed to their outer interface [29].

**Figure 6:**
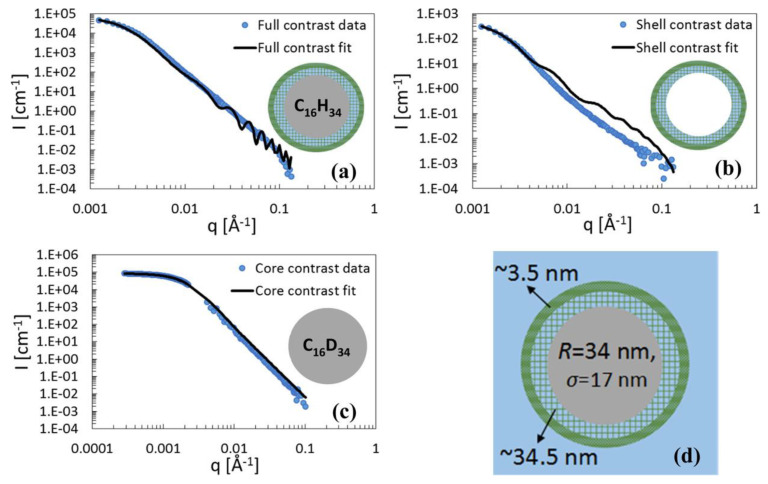
SANS patterns after background subtraction (blue circles) and core-two-shells model fits (solid line) of cellulose-coated n-hexadecane nano-emulsions, at three contrast settings: (**a**) full contrast, (**b**) shell contrast, and (**c**) core contrast; (**d**) scheme of the emulsion droplet according to the suggested core-two-shell model with the fitted model parameters. Reprinted (adapted) with permission from [32]. Copyright (2018) American Chemical Society.

Furthermore, SANS provide crucial insights into the structural features of polysaccharide-based systems with more complex dimensions, such as nanofibrils and nanogels. Nanofibrils of zwitterionic cellulose were combined with surfactants in solution and their colloidal properties were tested by SANS. The anionic and cationic surfactants sodium dodecyl sulfate (SDS) and dodecyltrimethylammonium bromide (DTAB), respectively, were used in their deuterated form to match the D_2_O solvent. At pH 7, free cellulose fibrils showed aggregation while by the addition of the cationic surfactant flocculation of the aggregates occurred. The nanofibrils were stabilized in their individual state by the anionic surfactant. A worm-like chain model revealed an elliptical cross-section with a major radius of 7.5 nm and a high persistence length that fell outside the SANS measurement Q-range [30]. The mechanical properties of cellulose fibers were tested in combination with SANS so that any correlation with their structure and morphology was revealed. The study used fibers produced by wet-spinning, dry-spinning, and dry-jet spinning. Using selective deuteration with deuterated solvents, a contrast between the amorphous and crystalline phases was achieved. Anisotropic 2D SANS patterns provided the ellipticity parameters of the meridional Bragg peaks [31]. Natural cellulose predesolved in 1-ethyl-3-methylimidazolium acetate was applied as a stabilizer of o/w emulsions. SANS and supporting ultra-small angle neutron scattering (USANS) experiments were performed at full contrast, i.e., hydrogenated components in D_2_O, shell contrast, i.e., matched oils to D_2_O and core contrast, i.e., matched cellulose to H_2_O/D_2_O mixture and deuterated oils. The fitting procedures were performed on all contrasts assuming that the structure was unaltered by solvent variation and oil deuteration. The data was modeled by a polydisperse oil core of 34 nm radius and a double shell. The shell thicknesses and volume fractions were 34.5 nm and 3% and 3.5 nm and 25%, respectively (Figure 6). The inner shell was considered a porous hydrogel as its scattering-contained contributions that were additional to those of the homogeneous shell. This structure would certainly have implications in nanocarriers for medical and nutrition compounds [32].

SANS on nano-sponges of crosslinked cellulose nanofiber hydrogels has revealed the expected network structures that was modeled by a combination of power-law scattering at low Q with the scattering of semidilute solution at high q with the extracted correlation length in the range of 1–10 nm [33]. O/W emulsions stabilized by cellulose fibrils have been modeled with a core-dense shell-hydrated shell model [34].

Electrostatic interaction between proteins and charged polysaccharides receives interest as it provides possibilities for self-organized complex and multifunctional structures. The anionic polysaccharide pectin was mixed with β-lactoglobulin at pH 4 (below the isoelectric point of the protein) to prepare coacervates. The SANS profiles showed a strong decreasing Q-dependence at low Q, which was attributed to the protein-induced aggregation of pectin at large length scales. At intermediate Q, a characteristic shoulder appeared as the protein/polysaccharide ratio increased from 10:1 to 30:1. The position of this shoulder Q* was translated to an average protein clustering domain size d = 2π/Q*. This size was in the order of 7–9 nm and was found to increase by the increase of the protein/polysaccharide ratio and salt content and by the decrease in the pectin’s charge density [35]. The internal morphology of thermally stabilized NPs from electrostatic complexes of BSA with chondroitin sulfate was probed by SANS. A three-level Beaucage model was used to fit the experimental data. High intermediate and low Q corresponded to BSA globules (3–5 nm), fractal clusters of BSA globules (15–30 nm), and scattering from the clusters’ arrangement within the NPs, respectively. Thermal treatment at pH 4.5 induced an enhancement of the clusters and a globule-to-coil conformation for individual BSA molecules. Upon an increase of pH to 7 (above the pI of the protein), the scattering profiles contain only two hierarchical levels revealing the morphology of a nanogel network. In addition, when salt was added at this pH, the three-level structure was revealed again pointing to the shrinking of the previously swollen nano-networks [23].

Chitosan that was grafted with several non-anionic polymers, was formulated into NPs by ionic crosslinking with tripolyphosphate. The resulting structures were identified as low-crosslinked nanogels. These NPs had higher mucus penetration ability than the ones of unmodified chitosan. Although the SANS signal-to-noise ratio was not strong, it was shown that the NPs SANS profiles were compatible with NPs 20–50 nm in size in contrast to the single chitosan-based copolymers [36]. The self-assembly of chitosan grafted with the hydrophobic polymer poly (methyl methacrylate) resulted to hierarchically structured NPs as it was revealed by SANS. A two-level Beaucage model provided R_g_ values for unimolecular or low aggregation number micelles and aggregates at 5 and 38 nm, respectively. The TPP-crosslinked NPs presented higher internal density. The findings supported the TEM and DLS results [37]. These works shed light to the internal morphology of self-assembled and crosslinked NPs based on modified chitosan which have strong implications on drug encapsulation, delivery and release.

### 3.2. Hydrogel and Nanocomposite Materials

Polysaccharide-based hydrogels have been extensively developed with regards to their use in medicinal applications (wound healing, tissue engineering, drug delivery), food sector, agriculture, water treatment, and other fields and hence the analysis of the structures of these systems in the length scales that SANS technique can cover is of great value.

Alginate, a polysaccharide commonly used in the food industry, often as a thickening agent, has also been proposed for biomedical and pharmaceutical applications and notably with promising potential regarding the management of metabolic disorders [38]. As part of the ongoing research [39,40], the SANS method has been employed for the study of calcium-alginate hydrogels enriched with glucose [8]. Preparation of (i) alginate solutions and (ii) hydrogels formed by crosslinking with Ca^2+^, in D_2_O solvent under the presence of different concentrations of deuterated glucose, resulted in distinct obtained data of the alginate chains due to contrast matching. Debye–Bueche function was applied for the analysis of scattered intensity in the low Q regime, thus allowing to obtain the characteristic size Ξ of inhomogeneities, induced by the presence of glucose. It was shown that Ξ increased under the addition of glucose (up to 465 ± 20 Å at 45 wt.%) in comparison to 320 ± 20 Å when no glucose was added. Furthermore, overlap of the scattering curves of solutions and gels at 30 and 45 wt.% glucose content showcased that gelation did not induce structural changes at measured length scales of up to 465 Å.

**Figure 7:**
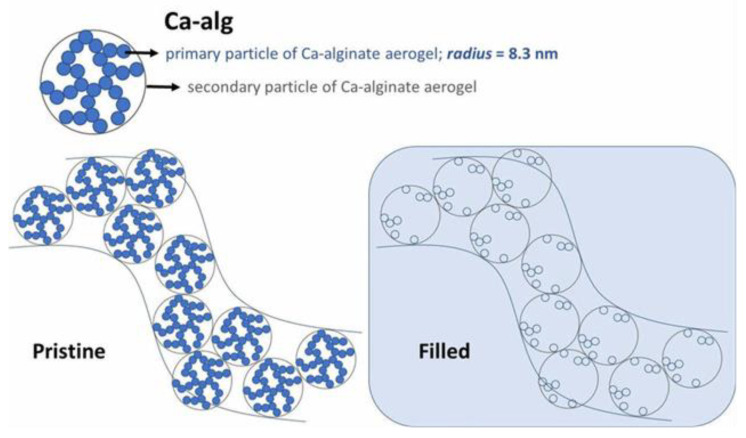
The proposed nanoscale structure of native Ca-alginate (Ca-alg) aerogel. The panel labeled “Filled” denotes filling with a contrast matching agent (H_2_O–D_2_O mixture of 46 wt.% H_2_O—54 wt.% D_2_O) in SANS. Reprinted (adapted) with permission from [41]. Copyright (2021) American Chemical Society.

Calcium alginate has also been studied via SANS in the form of aerogels [41]. Beaucage model, that is generally used for the analysis of regions that are expressed by Guinier and Porod approximations, was applied for fitting different regions of the SANS curves. Particle radii exported by the scattering curves (modeling network building blocks as spheres) showcased good agreement with those calculated by skeletal density and N_2_-sorption data and based on complementary SEM imaging and the proposed morphology of the aerogels as hierarchical primary and mass-fractal secondary particles, the authors were able to reproduce the nanoscale structure of the aerogels (Figure 7).

**Figure 8:**
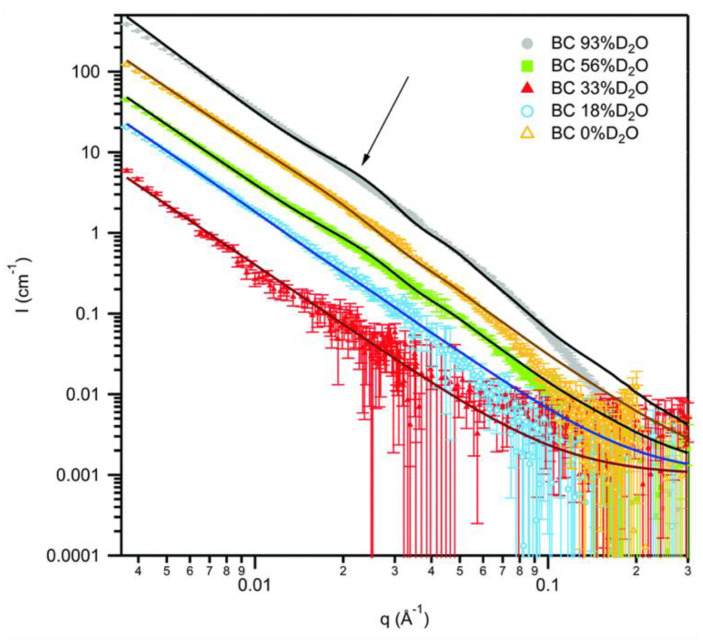
SANS patterns from solvent contrast variation experiments on bacterial cellulose hydrogel. Dots represent experimental data whereas the solid lines correspond to the best global fits obtained using the power-law plus core-shell cylinder model. The shoulder feature detected in the experimental data, which results from the SLD contrast between the different phases, is indicated with arrow. Reprinted (adapted) with permission from [45]. Copyright The Royal Society of Chemistry 2016.

Cellulose, the most abundant native polysaccharide on earth, has been widely investigated by SANS in the framework of various current and feature applications [42,43,44,45,46]. A variety of structural models ranging between parallelepipedons to cylinders with polydisperse radii have been proposed for the interpretation of the experimental data. Figure 8 shows the experimental SANS contrast variation data of bacterial cellulose together with the fitting results of a structural model combining a core-shell cylinder with polydisperse radius and a power-law contribution to low Q, as described in detail in [45]. The contrast matching point of bacterial cellulose was estimated to correspond to 34.2% D_2_O content of the solvent. The SLD of cellulose increases with the D_2_O content of the solvent (Figure 3), which is due to the exchange of hydrogen from the labile hydroxyl groups with deuterons from the solvent. A detailed discussion of the effects of the H/D exchange process on the approach to interpreting the experimental data based on SLD variation can be found in [45].

In a recent publication [47], the authors conducted SANS experiments to study the effect on dilute acid pretreatment (DAP) on model hemicellulose-cellulose composites. Hemicellulose (glucomannan and xyloglucan) stock solutions were prepared in D_2_O and diluted in deuterated growth media following the introduction of D_2_O adapted A. xylinus A. xylinus sucrofermentans inoculum as part of the preparation of the composites. The DAP approach resulted through the addition of dilute acid and heat treatment. The R_g_ of glucomannan-cellulose composites decreased after pretreatment compared to the native state, similarly to the obtained results of native cellulose, with the authors ascribing it as result of expulsion of water from the highly hydrated cellulose macrofibril due to the heat treatment. On the contrary, the R_g_ of xyloglucan-cellulose composites was found to decrease to an insignificant degree between the two states, showcasing a different behavior.

In another study [48], the effect of different surfactants (hexaethylene glycol mono-n-dodecyl ether (C12EO6), sodium dodecyl sulfate (SDS), cocamidopropyl betaine (CapB), and dodecyltrimethylammonium bromide (DTAB)) on TEMPO-oxidized cellulose nanofibril (OCNF) suspensions was analyzed. Data were fitted based on OCNF modeled as rod-like particles with elliptical cross sections. In the case of mixtures of 1 wt.% OCNF and (anionic) SDS micelles, study of the fibrils allowed by contrast matching using d-SDS and D_2_O revealed increased attraction compared to OCNF without micelles, while micelle-micelle electrostatic repulsions also appeared to decrease in the counterpart analysis. Mixtures of OCNF with zwitterionic (CapB) did not appear to experience fibril-micelle interactions and SANS data analysis fitted as sum of contributions of the micelles and the nanofibrils was adequate without the requirement of other adjustments.

Oxidized cellulose nanofibrils (OCNF) in the presence of silica nanoparticles (SiNp) of two different diameters (5 nm—SiNp5, 158 nm SiNp158) as non-interacting fillers have been investigated using the SANS technique in a recent publication [49]. The effect of SiNps on the structural properties of the OCNF network was enabled via the contrast matching of the particles through the continuous phase of 60 vol% D_2_O/40 vol% H_2_O mixture. Fitting of the scattering intensity data was applied in the intermediate and high Q region in the basis that interfibrillar interactions are not detectable as opposed to the low Q range. Comparison of SANS patterns of 1 wt.% OCNF network and 1 wt.% OCNF network with added SiNps of two different concentrations (1 wt.%-SiNp5, 2.5 wt.% SiNp5, 1 wt.%-SiNp5, 2.5 wt.% SiNp158) revealed that the Silica particles did not affect significantly the structure of the network.

Stimuli-responsive properties such as thermo-responsiveness are valuable for the development of advanced biomedical systems. Thermoresponsive hydrogels synthesized by the adsorption of PDMAEMA (poly(2-(dimethylamino)ethyl methacrylate))-b-PDEGMA (poly (di (ethylene glycol) methyl ethermethacrylate)) block copolymers on oxidized cellulose nanocrystals (TO-CNCs) were investigated by the SANS technique in terms of the study of the polymer behavior on the TO-CNCs surfaces [50]. SANS data for system of 1:1 mass ratio of TO-CNCs/block copolymer revealed significant increase in the scattering intensity in the low Q-region as compared to the intensity measured on TO-CNCs (without the presence of block copolymers) suggesting effect of polymer chains on the formation of CNCs bundles, while heating of the sample upon the copolymer’s lower critical solution temperature (LCST) led to the formation of dense aggregates of TO-CNCs as shown by the measured Q decay.

Time-resolved small angle scattering is often applied for the analysis of structural changes and rheological properties of hydrogel systems. A recent study focused on the effect of photopolymerization synthesis method on the kinetics of the network morphology of Starch hydrogels [51]. Evolution of the network formation was captured through repeated alternating steps of very small-angle neutron scattering (vSANS) measurements and exposure of samples to UV light. The exported scattering profiles were fitted by an equation that combines a Porod term which is dominant in low Q-range and an Ornstein–Zernike Lorentzian term corresponding to the scattering of the polymer chains in the fluid scale that was chosen by the authors for vSANS analysis due to relevance with small-angle oscillatory shear rheology measurements that they also conducted for the observation of the gelation of the hydrogels on bulk scale. Additionally, the two terms include respective exponents, the Porod exponent that can indicate potential clustering or Gaussian swollen chains formation and the Lorentzian exponent that is relevant to polymer–solvent interactions. Amongst other measurements, kinetics of hydrogels formed by neutral starch nanoparticles (SNPs), were compared to those based on charged (cationic and anionic) SNPs. Prior to the photopolymerization, the Lorentzian exponent of both (−)SNP and (+)SNP hydrogels were found to be lower than that of neutral ones, showcasing charge-induced swelling. An increase in the fluid exponent upon photopolymerization and subsequent slow decrease was attributed to the attraction of SNPs during crosslinking and deswelling occurred as compensation for the reduction of interparticle repulsion.

Rheo-small-angle neutron scattering (Rheo-SANS) experiments were conducted by researchers using stress-controlled rheometer, in order to investigate the rheological behavior of polymer-like micelles (PLMs) undergone oscillatory shearing [52]. More specifically, time-resolved rheo-SANS was applied for the investigation of the strain shifts’ impact on the structure of PLMs. Obtained 2D scattering patters were deduced to 1D I(q) plots through a ‘fixed’ q* corresponding to the segmental length of micelles over two azimuthal angles (φ). Scattering results showcased that the micellar structure was not affected under oscillatory shearing. Moreover, scattering intensity as measured at different stress phase angles ψ = 0, π/4, 2π/4 under zero recoverable strain appeared to be isotropic (independent of the measured angle) in contrast with the results of the maximum recoverable strain where intensity peaks at = 90° and 270° angles highlight anisotropic scattering.

Horkay and Douglas [53] studied polyacrylic acid (PAA) and DNA gels’ osmotic characteristics associated with the many-body interactions present in such systems as polyelectrolyte gels. Gel volume (quantified as gel swelling degree, 1/φ) was found to gradually decrease as the concentration of Ca2^+^ ions (originated from CaCl_2_) was increasing, up to a critical concentration (in PAA at approximately 1 mM CaCl_2_ and in DNA at approximately 0.3 mM CaCl_2_) where volume transition was indicated.

Ultra-small angle neutron scattering (USANS) (q ranges: 0.006–0.6 Å^−1^/7.63 × 10^−4^–0.65 Å^−1^) experiments were performed for the investigation of dual transient networks consisting of worm-like micelles (WLMs) of oppositely charged surfactants (potassium oleate/n-octyltrimethylammonium bromide) with polysaccharide chains of hydroxypropyl guar (HPG) [54]. USANS curves were obtained at fixed surfactant concentration (2.5 wt.% potassium oleate/0.8 wt.% n-octyltrimethylammonium bromide) and increasing total HPG concentration ranging from 0 to 2% wt.%. High q-region parts of the scattering curves of the three samples appeared to overlap suggesting that the cylindrical structure of WLMs was not affected by the addition of the polysaccharide, while in the lowQ region scattering intensity increased in dependence with the polymer concentration indicating microphase separation.

### 3.3. Self-Assembly and Macromolecular Conformation

The small-angle neutron scattering (SANS) technique offers valuable insights into exploring self-assembly processes within colloids and amphiphilic systems. Additionally, it plays a crucial role in investigating the mechanisms of phase transitions and aggregation kinetics across a diverse range of nanomaterials and biomaterials. SANS provides a precise methodology for identifying order-disorder transitions and for following changes of macromolecular conformations in various nanostructured material systems, including those based on natural and chemically modified polysaccharides. In the field of nanomaterials and biomaterials the impactful applications of small-angle scattering involve studying assembly processes influenced by physico-chemical variables or internal/external perturbations, such as concentration, pH, ionic strength, and temperature, among other factors.

**Figure 9:**
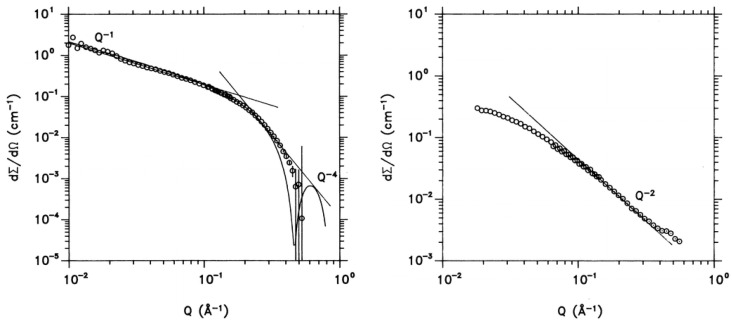
**Left**—Macroscopic scattering cross-section dΣ/dΩ of fragmented cinerean in H_2_O compared with the calculated form factor (**left**). The typical Q^−1^ dependence for rod-like particles as well as the Porod Q^−4^ law are indicated by straight lines. **Right**—Scattering pattern from cinerean in aqueous 0.4 N NaOH solution. From both the Q^−2^ dependence (indicating a random coil conformation) and the low Q or Guinier regime the radius of gyration R_g_ of the coil can be consistently deduced. Reprinted with permission from [55]. Copyright (1996) American Chemical Society.

Utilizing the SANS technique provides valuable insights into conformational characteristics of polysaccharides. Here, pioneering work on cinerean [55], a microbial b-(1-3)(1-6)-D-glucan produced by the fungus *Botrytis cinerea*, may be mentioned. The native cinerean is a worm-like chain with a persistence length. It was found combining SANS and SAXS, that in H_2_O and D_2_O solutions, fragmented cinerean has a rod-like, triple-helical conformation, which accounts for rigidity of the rods. In solutions with NaOH the cinerean multihelix disentangles into its single strands, which have a random coil conformation. The transition between helix and coil was examined with SANS (Figure 9).

SANS intensity originates from the existence of the cross-linking points as it was concluded from studies of the gel-to-sol transition in *k*- or *i*-carrageenan gels in D_2_O [56,57]. Carrageenan belongs to a family of algal polysaccharides and its solution exhibits a thermo-reversible sol-gel transition. k-carrageenan has one sulfate group for every monomer, while i-carrageenan presents two sulfate groups; therefore, gelation is promoted under the existence of cations, such as Na^+^ or K^+^, since the cation neutralizes the Coulomb repulsion force between the polymer chains in the cross-link point. The SANS revealed that the physically cross-linked aggregates of *i*-carrageenan gels are smaller than those of the *k*-carrageenan gels and that has consequences on the macroscopic rheological property of carrageenan gels [58].

Mannan polysaccharides obtained from the different sources have been investigated and compared [59]. The experimental evidence suggests that the mannan exopolysaccharide from *P. arcticus* bacterium is structurally characterized by rigid-rod regions assuming a 14-helix-type conformation. In a similar way, SANS was employed to compare the structural features of deuterated chitosan produced from the filamentous fungus *Rhizopus oryzae*, which was cultivated with deuterated glucose in aqueous medium with a structure of non-deuterated counterpart [60]. SANS analysis with two structural levels (power law exponent at high Q region and cylinder form factor at low Q region) shows very similar structures of deuterated and hydrogenated chitosan. The most abundant radii of the protonated and deuterated chitosan fibers were 54 Å and 60 Å, respectively, but the protonated sample had a broader distribution of fiber radii.

**Figure 10:**
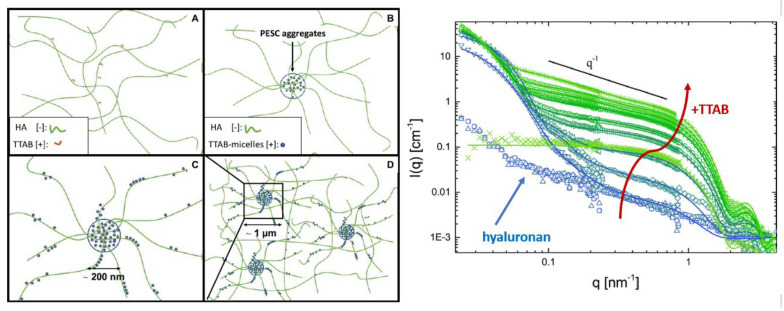
**Left** side: Schematic overview of the structural impact of TTAB on HA. (**A**) HA network with loosely attached TTA molecules (c < 0.5 mM). (**B**) Globular aggregates within HA/TTA micelle cluster (0.5 mM < c < 4 mM). (**C**) Cylindrical complexes are formed by HA and TTA micelles throughout the sample (c > 4 mM). (**D**) Zoom out of the situation depicted in (**C**). **Right** side: SANS intensities as a function of the scattering vector q of selected HA/TTAB mixtures upon increase of TTAB concentration (from blue triangles—pure HA to green crosses—pure TTAB) in 150 mM PBS in D_2_O at 25 °C. Adapted with permission from [9]. Copyright (2020) American Chemical Society.

Moreover, the SANS method is extensively used for investigating the polyelectrolyte-surfactant assemblies including systems composed of polysaccharide-based polyelectrolytes [9,61,62,63,64,65]. Grillo et al. [61] characterized the Pluronic F127 micellar solutions in the presence of hyaluronic acid (HA) in semi-dilute regime. Applying SANS, it has been shown that addition of HA (with HA volume fractions above 1% and with molecular masses of 12 and 300 kDa) have no effect on the size and shape of F127 micelles. However, the change of hyaluronic acid chain conformation from stretched to coil due to the increase in salt concentration induced the formation of micellar clusters that further organize liquid crystalline phase. Such behavior is reinforced by increasing HA concentrations or molecular weight as well as by transitioning from monovalent to divalent cations such as Ca^2+^. The electrostatic assembly of hyaluronic acid (HA) and the surfactant tetradecyltrimethylammonium bromide (TTAB) exhibits a distinctive structural arrangement, differing significantly from the densely packed micellar systems observed in other polyelectrolyte-surfactant complexes [9]. The authors propose that the association behavior resembles local coacervate formation, with the unique properties of HA, particularly its high stiffness, playing a governing role in this process. The stiff backbone and the charged groups that are in close proximity to this backbone and the ability to form a network in solutions, which remains relatively unaffected by the introduction of a surfactant, facilitate the binding of TTA micelles only without significantly disrupting the existing HA network. SANS demonstrates the formation of large globular superstructures as a result of addition small amount of TTAB (Figure 10). These structures are constructed hierarchically from a local thread-like arrangement of TTA micelles along the stiff HA chains. These globular domains, with radii ranging from 60 to 100 nm, consist of 500 to 700 TTA micelles and are notably water-rich. Importantly, they do not increase in size or quantity with further addition of TTAB. Instead, the extra TTA micelles form additional thread-like complexes outside the existing large globular domains. Applying contrast variation SANS provide deeper insight into the structural arrangement. By matching scattering from the TTA micelles it has been shown that HA network remains relatively unaffected upon the increasing surfactant concentration and, especially, it is not more densely packed in the observed large aggregates than outside of them.

Polyelectrolyte-surfactant complex system composed of cationic cellulose-based polyelectrolyte JR400 and anionic surfactant sodium monododecyl phosphate exhibits drastic changes in viscosity caused by changing solution pH. Therefore, changes of the mesoscopic structure of the system were elucidated with small-angle neutron scattering and neutron spin-echo spectroscopy. Data were modeled as a superposition of free polyelectrolyte (as thin cylinder with the same size as found for the pure PE), aggregates, and a power law exponent. Obtained results suggest that viscosity increase at pH 7 is caused by formation of mixed rod-like PE/surfactant aggregates which interconnect approximately by 3 polyelectrolyte chains. These aggregates are dissolved at pH 12, where the surfactant bears 2 charges and as result is less prone to aggregate [63]. In another recent study [64] a novel approach to control the charge ratio phase between cationic hydroxyethyl cellulose (cat-HEC) and oppositely charged surfactant sodium dodecyl sulfate (SDS) at fixed concentrations has been established. Charge ratio between the components was tuned by applying a homologous series of cat-HEC with a different degrees of side chains charge modification. Self-assembly leading to complex formation was proved by comparison with not charged modified HEC polysaccharide. For the better examination of formed nanostructures contrast variation SANS was performed applying hydrogenated and deuterated surfactant. Scattering consistent with thin rods with an average radius of ca. 7.7 Å and length of ca. 85 Å was observed for all catatonically modified polysaccharides. For the charge-modified polymers, interactions with SDS were evident in contrast to neutral polymer, and the radius of the formed complexes grew up to ca. 15 Å with an increasing charge ratio between polysaccharide and surfactant, Z. Additionally, an increase in scattering intensity at low Q region shows the formation of large polymer network structures as Z tends towards 1.

Small-angle neutron scattering (SANS) is a powerful technique extensively utilized for characterizing cellulose, a fundamental component of plant cell walls, and an essential biopolymer. SANS provides valuable insights into the structural features of cellulose and derivatives at the nanoscale. SANS data collected on solutions of different types of nanocellulose revealed their fibrillar morphology [66] and delivered the dimensions of nanocellulose when modeled using the rigid parallelepipedon form factor, based on the observed scattering features and under the assumption that the fiber length was much larger than characteristic lengths of cross section. SANS could also be utilized for monitoring cellulose dissolution and the impact of chemical treatment [67,68,69,70,71]. Microbial cellulose (MC) produced by *Acetobacter xylinum* is notable for its remarkable water content. Investigation of the hierarchy within the bacterium body and the local non-crystalline structure by SANS and USANS allowed by polarization analyses revealed a domination of capillarity, linked to hierarchically preserved non-crystalline domains, in the water uptake mechanism. In contrast, as a benchmark, the high-water absorption observed in PNIPAm gels is associated with a tight hydration mechanism onto a monomer unit through hydrogen bonds [72]. Raghuwanshi and co-authors [73] investigated dissolution of cellulose in ionic liquid 1-ethyl-3-methylimidazolium acetate (EMIMAc) by contrast variation SANS in combination with SAXS method. Two experiments were performed: dissolution of hydrogenated cellulose in deuterated IL and oppositely deuterated cellulose was dissolved in hydrogenated IL. Study emphasizes that the strong binding of at least one acetate ion to each anhydroglucose unit occurs. EMIMAc serves as an effective solvent for cellulose as it effectively charges the cellulose chains, preventing their clumping in the solution. Mild alkaline treatment on the structure and enzymatic hydrolysis of waxy maize starch, investigated through small-angle neutron scattering (SANS) across an extended q-range, indicate that the enzymatic activity of both the 0.1% (*w*/*v*) and 0.5% (*w*/*v*) alkaline solutions primarily takes place on a smaller length scale, specifically affecting starch double helices and crystallites. In contrast, larger-scale structural features such as lamellae, growth rings, and blocklets remain largely unchanged [74]. Additionally, rheological properties of semidilute solutions of sodium carboxymethyl cellulose (NaCMC) have been explored. The study involved varying the degree of substitution (DS) and concentration of NaCMC and examining its behavior in diverse solvent media to selectively analyze the distinct roles played by electrostatic and hydrophobic interactions. These media included salt-free water (with long-ranged electrostatic interactions), 0.5 M aqueous NaCl (involving screened electrostatics), and 0.5 M aqueous NaOH (involving screened electrostatics and diminished hydrophobic interactions). In summary, SANS data indicate that hydrophobic interactions minimally influenced polymer conformation. Through variations in DS, ionic strength, and pH, the study demonstrates the capability to tailor NaCMC–solvent interactions, effectively controlling the electrostatic and hydrophobic effects on the solution rheology. Thus, DS reducing leads to reduced solubility, partial aggregation, and eventual gelation. In salt-free and 0.5 M NaCl solutions, NaCMC with DS ≃1.2 exhibits hydrophilic polyelectrolyte and neutral polymer behavior in a good solvent, respectively. Decreasing DS to ≃0.7–0.8 results in hydrophobic behavior in both media, forming weak gels at high concentrations. In 0.5 M NaOH, solutions with different DS show identical viscosities when plotted against the overlap parameter, suggesting the solubilization of unsubstituted cellulose blocks [75].

Besides that, SANS enables the investigation of cellulose phase behavior under different conditions, aiding in the applying cellulose-based materials in diverse fields such as biomaterials, nanotechnology, and sustainable manufacturing. Corroboration of SANS with other methods underscores phase behavior of concentrated rod-like cellulose nanocrystals in aqueous suspension revealing the start of cellulose nanocrystal alignment and a decreasing distance between the cellulose nanocrystals with increasing concentration. At 2 wt.%, the first correlation peak in the SANS-determined structure factor treated by Lorentzian function corresponded to a correlation distance of ~80 nm [76]. Another relevant study [77] was dedicated to investigation of phase behavior of aqueous dispersions of acetylated cellulose nanocrystals (CNC-AA) with acetyl ester surface functional groups. Small-angle neutron scattering was employed to measure the expansion of fractal structures upon increasing concentration. The parallelepiped form factor model with a rectangular cross-section, averaged by Gaussian polydispersity over all space orientations was found to best fit the scattering data. The results suggest a two-dimensional assembly characterized by short-range order in a plate-like assembled geometry. Furthermore, recently Mao [78] and coworkers investigated isotropic–nematic (I–N) transitions in cellulose nanocrystal (CNC) suspension and self-assembled structures in the isotropic and nematic phases. Analysis of 2D and 1D scattering profiles indicate that the nematic phase contains more populations of larger particles. Fitting of scattering curves suggest that CNC particles form stacks with interparticle distance of 37 nm in both phases. Also, it has been shown that applying magnetic field was able to induce a preferred orientation of CNC stacks in the nematic phase, with the stack normals being aligned with the field and decrease of the fraction of oriented CNS within the relaxation.

**Figure 11:**
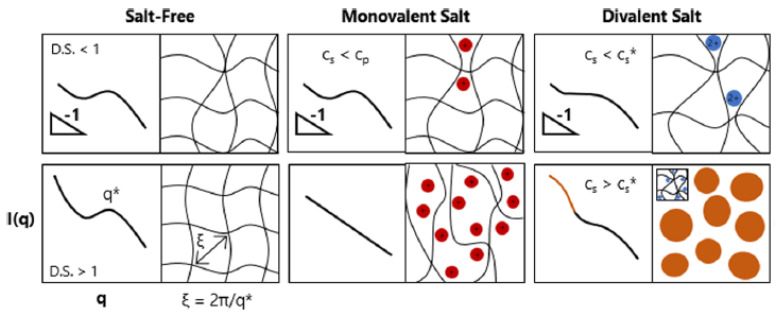
Representation of semidilute NaCMC solutions with varying degree of substitution (D.S. < 1 top row and D.S. > 1 bottom row) and added salts (salt concentration, c_s_ below and above polymer concentration, c_p_ for monovalent salts; and below or above phase boundary concentration c_s_* for divalent salts). Boxes on the left are the SANS profile and boxes on the right are the physical interpretation of the solution. Reprinted with permission from [79]. Copyright (2020) American Chemical Society.

Sharrat and coworkers [79] explored both the conformation and phase behavior of sodium carboxymethyl cellulose (NaCMC) in the presence of mono- and divalent salts. Their investigation focused on analyzing the characteristic polyelectrolyte correlation peak, examining its presence, position, and calculating the correlation length between polysaccharide chains and following the power law of the scattering profile. The observed results revealed charge screening induced by addition of monovalent NaCl and divalent Mg^2+^ which does not interact specifically with NACMC. Additionally, the introduction of divalent cations Ca^2^⁺, Zn^2^⁺, and Ba^2^⁺, which interact specifically, resulted in an amplified screening effect on the correlation peak. Beyond the phase boundary, excess scattering at low Q suggested the formation of 20–40 nm clusters. This behavior was linked to a reduction in charge density along the chain, promoting interchain association and the formation of multichain domains, leading to visible turbidity. Representation of semidilute NaCMC solutions in the absence of salt and in the presence of mono- and divalent salts based on SANS profiles is showcased in Figure 11.

### 3.4. Nanostructured Plant and Food Materials

**Figure 12:**
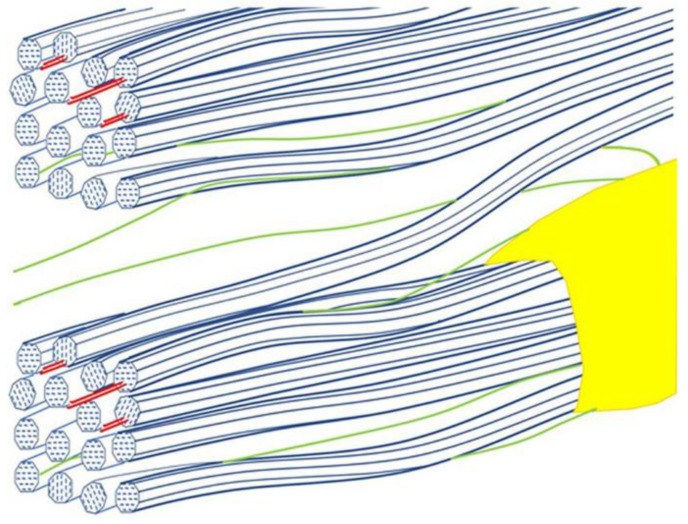
Diagrammatic view of a portion of the spruce cell wall, with two cellulose macrofibrils (blue) containing xylan (red) that is partly bound to cellulose surfaces. The macrofibrils are separated by a matrix composed largely of glucomannan (green) and beaded lignin (yellow). Two macrofibrils are shown bridged by a single microfibril, suitably positioned to transmit shear load between the macrofibrils if their position within the larger-scale structure leads to their axial stress being unequal. Within each macrofibril the microfibrils are shown as held together by non-covalent forces but the right-handed microfibril twist prevents attachment zones from being axially continuous [82].

The study of the nanostructure of plant cell walls is of emerging research interest and SANS method is beneficial due to its’ characteristics in order to reveal information about the complex morphology that is mainly consisted of cellulose-based microfibrils organized as bundles, lignin, and pectin polymers, hemicelluloses like xylan and glucomannan as well as high water content. The structure of part of spruce cell wall is displayed in Figure 12.

Water-accessibility of nanoscale pores present in the fibrillar plant (spruce) cell walls was analyzed by time-resolved SANS [80]. Amongst the three parameters that constitute the model that was selected for the analysis of the data and include the contribution of cellulose microfibrils, the aggregation of the microfibrils and the power-law scattering by large pores and cell lumina, the only scaling factor that was found to alter over time was that of the first term, indicating that the water diffusion was homogenous and independent of the distances between the microfibrils.

In another relevant study, the authors employed time-resolved SANS for the investigation of structural changes on microfibril bundles of spruce cell wall induced by water loss during drying process [81]. Similarly, to the study mentioned above, model fitting of the scattering intensity patterns included the terms corresponding to the scattering intensities provided by the microfibrils, microfibril bundles, and lumina. The observed drying kinetics were similar to those known for porous materials, dominated by two distinct regions: constant rate period (CRP), where the drying rate is rather steady, and falling rate period (FRP), where drying rate starts to decrease. Fibril-to-fibril distance was found to decrease during the FRP as a result of the water loss between the microfibrils, while at the prior CRP, water removal seemed to correspond to the cell lumina without significant effect to the cell wall nanostructure.

The impact of tension on the nanostructure of spruce wood was the subject of another study [82]. SANS measurements obtained the following results: initially, under tensile strain and at relaxation phase signified that the characteristic center-to-center spacing of microfibrils (3.1 nm) that was extracted by the fitted major coherent peak of the scattering intensity plot was not notably affected under tension.

**Figure 13:**
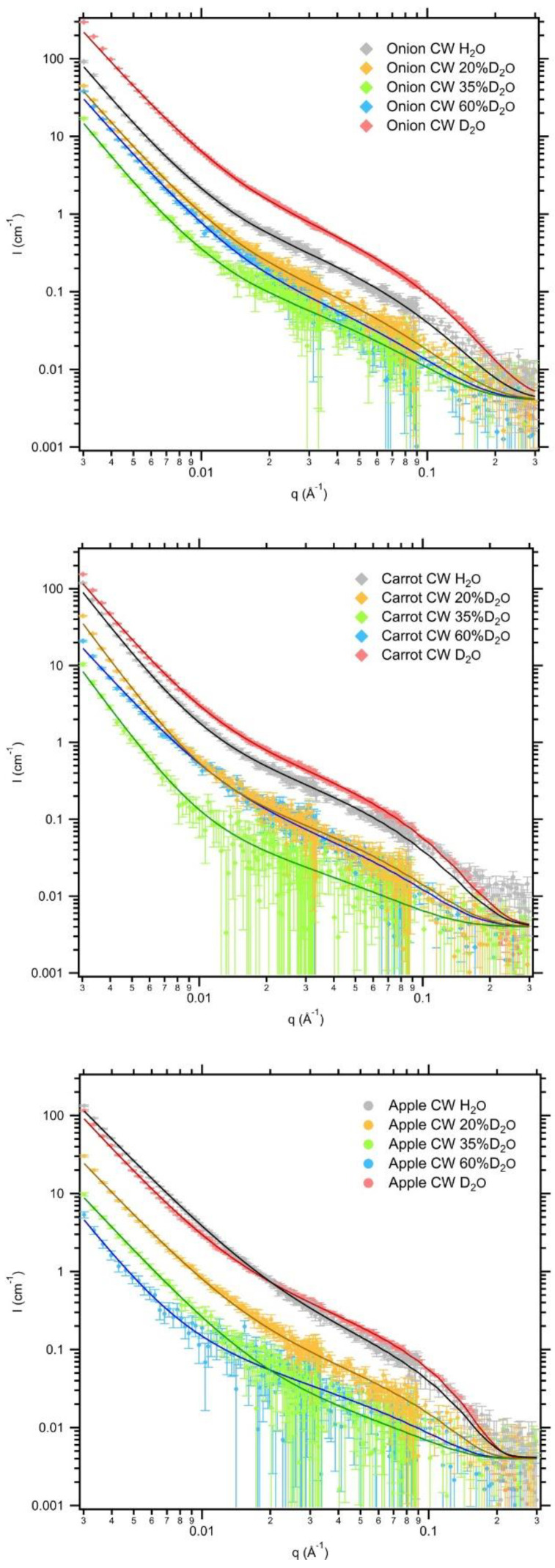
Contrast variation SANS and fitting curves on cell walls extracted from onion carrot and apple at different D_2_O volume fractions in D_2_O/H_2_O solvents. Reprinted from [83], Copyright (2020), with permission from Elsevier.

The influence of the presence of pectin and its interactions with cellulose in the plant cell walls was the subject of a recent publication [83]. SANS contrast variation was employed at 0, 20, 35, 60, and 100% *v*/*v* D_2_O in D_2_O/H_2_O mixed solvent and the core-shell model [84] was used to simultaneously fit the data (Figure 13). Contrast variation curves that took H/D exchange into account showed that contrast matching of pectin occurs at 33% D_2_O. SANS method was used for the characterization of pectin-rich cell wall suspensions from apple, carrot and onion and the data were fitted via the core-shell model that encompass a power-law term that corresponds to large-scale structure, a term representative of the core-cell wall structure and a third term that covers incoherent background. As showcased by complementary SAXS measurements, the onion cell wall was the one with the smallest cellulose microfibril cross-section amongst the samples, indicating relatively higher pectin content. Based on the decreased solvent H/D exchange within the microfibril shell estimated for the onion samples (ca. 20% compared to ca. 80% for carrot and ca. 90% for apple) and the cellulose volume fraction (ca. 0.8 for onion, ca. 0.4 for apple and carrot) the authors deduced that pectin has a densification effect by reducing the restrained water within the cellulose paracrystalline fraction [83].

Cellulose nanocomposites have been proposed by researchers over a wide range of applications including three-dimensional printing, optoelectronics [85], electromagnetic shielding [86] and regenerative medicine [87] to name a few. In this context, PMMA-cellulose nanocomposites, referred to as “transparent wood” due to their remarkably high optical transmittance, were prepared [88]. As part of the process, lignin and part of the hemicelluloses were removed and the structure of the delignified wood substrate with deuterated PMMA filling the pore space (DelW/D-PMMA) was analyzed with SANS. Scattering peak at 0.09 Å^−1^ was observed in the anisotropic scattering intensity plot and was attributed to the distribution of the MMA between individual microfibrils. This hypothesis was confirmed by following SANS data where PMMA matrix was contrast-matched with cellulose and the peak was no longer present.

The use of nanocomposites composed of nanocellulose in combination with graphene for the development of conductive paper is another promising research subject and the use of surfactants as stabilizers has been proposed as an alternative to other more prevalent preparation methods [89]. The behavior of surfactants’ solutions, that are known to self-assemble into aqueous phrase, was examined through SANS measurements and compared with solutions at the same surfactant concentrations and incorporated reduced graphene oxide (RGO). Contrast matching was applied and following scattering profiles were fitted by employing ellipsoidal (for DDAP/DTAB) and spherical (SDS) models. Scattering intensity peaks were less pronounced in surfactant/RGO solutions indicating the effect of RGO on the constraint of intermicellar interactions.

The complex structure of oleosomes has been thoroughly studied over a series of SANS experiments [90]. Guinier radius Rg (1380 Å) as well as shell thickness t (9 nm) were determined by polydisperse core-one shell model that incorporates the parameters of the thickness and SLD of the shell and the radius, polydispersity, and SLD of the core. Additionally, the scattering profiles of oleosomes encapsulated with pectin by electrostatic deposition at pH 4 were obtained at different D_2_O concentrations (0, 12 and 40%) and the encapsulation of pectin was highlighted by the increase in the shell thickness (t = 198 Å).

In conclusion, the abundance of published studies of SANS-analyzed cellulose/plant nanostructures [91,92,93,94,95,96,97] demonstrates the importance of the technique for deeper understanding of these systems and future development of novel environment-friendly materials.

## 4. Conclusions and Future Perspectives

This review presented the state of the art in the SANS studies on polysaccharide materials which are very important for the research and applications in food science, biomaterials, and natural materials. The theoretical background of SANS and the ideas on contrast matching/variation are explained so that the discussion on the several uses of SANS in the recent literature is complete. The areas of nanoparticulate systems, hydrogels and nanocomposites, self-assembly, and plant materials are covered as they are currently in the spotlight of polysaccharide research. The potential of the use of SANS for advanced characterization and optimization of novel polysaccharide materials is revealed as these materials may be organized in multiple length scales and in combination with other compounds may form nanostructures of various forms. Such structural organization is responsible for the unique mechanical properties and thus also for the textural and rheological properties of such materials, which have an impact on food and ingredients, but also on energy-related topics such as biofuels.

## Figures and Tables

**Table 1:** Scattering lengths and cross sections of coherent and incoherent neutron scattering for nuclei relevant for synthetic and natural hydrocarbons [18].

Atomic Nucleus	b_coh_ [× 10^−13^ cm]	b_incoh_ [× 10^−13^ cm]	s_coh_ [× 10^−24^ cm^2^]	s_inc_ [× 10^−24^ cm^2^]
^1^H	−3.74	25.27	1.758	80.27
^2^H (D)	6.67	4.04	5.592	2.05
^12^C	6.65	0.0	5.550	0.00
^14^N	9.37	2.00	11.01	0.50
^16^O	5.80	0.00	4.232	0.00
^31^P	5.13	0.20	3.307	0.005
^32^S	2.80	0.00	0.988	0.00

## Data Availability

Not Applicable.
